# In Metabolic Engineering of Eukaryotic Microalgae: Potential and Challenges Come with Great Diversity

**DOI:** 10.3389/fmicb.2015.01376

**Published:** 2015-12-15

**Authors:** Javier A. Gimpel, Vitalia Henríquez, Stephen P. Mayfield

**Affiliations:** ^1^Chemical and Biotechnology Engineering Department, Centre for Biotechnology and Bioengineering, Universidad de ChileSantiago, Chile; ^2^Instituto de Biología, Pontificia Universidad Católica de ValparaísoValparaiso, Chile; ^3^Division of Biological Sciences, California Center for Algae Biotechnology, University of California, San DiegoLa Jolla, CA, USA

**Keywords:** microalgae, metabolic engineering, transformation, carotenoids, biodiesel, biohydrogen, PUFA, photosynthesis

## Abstract

The great phylogenetic diversity of microalgae is corresponded by a wide arrange of interesting and useful metabolites. Nonetheless metabolic engineering in microalgae has been limited, since specific transformation tools must be developed for each species for either the nuclear or chloroplast genomes. Microalgae as production platforms for metabolites offer several advantages over plants and other microorganisms, like the ability of GMO containment and reduced costs in culture media, respectively. Currently, microalgae have proved particularly well suited for the commercial production of omega-3 fatty acids and carotenoids. Therefore most metabolic engineering strategies have been developed for these metabolites. Microalgal biofuels have also drawn great attention recently, resulting in efforts for improving the production of hydrogen and photosynthates, particularly triacylglycerides. Metabolic pathways of microalgae have also been manipulated in order to improve photosynthetic growth under specific conditions and for achieving trophic conversion. Although these pathways are not strictly related to secondary metabolites, the synthetic biology approaches could potentially be translated to this field and will also be discussed.

## Introduction

Microalgae, defined as a polyphyletic group of unicellular photosynthetic eukaryotes, are among the most ancient and diverse organisms on the planet. There are at least 40,000–70,000 species belonging to nine different phyla. Additionally, some estimates propose that there could be up to eight times the amount of undiscovered or unclassified species (Norton et al., 1996; Bhattacharya et al., 2004; Guiry, 2012).

**FIGURE 1:**
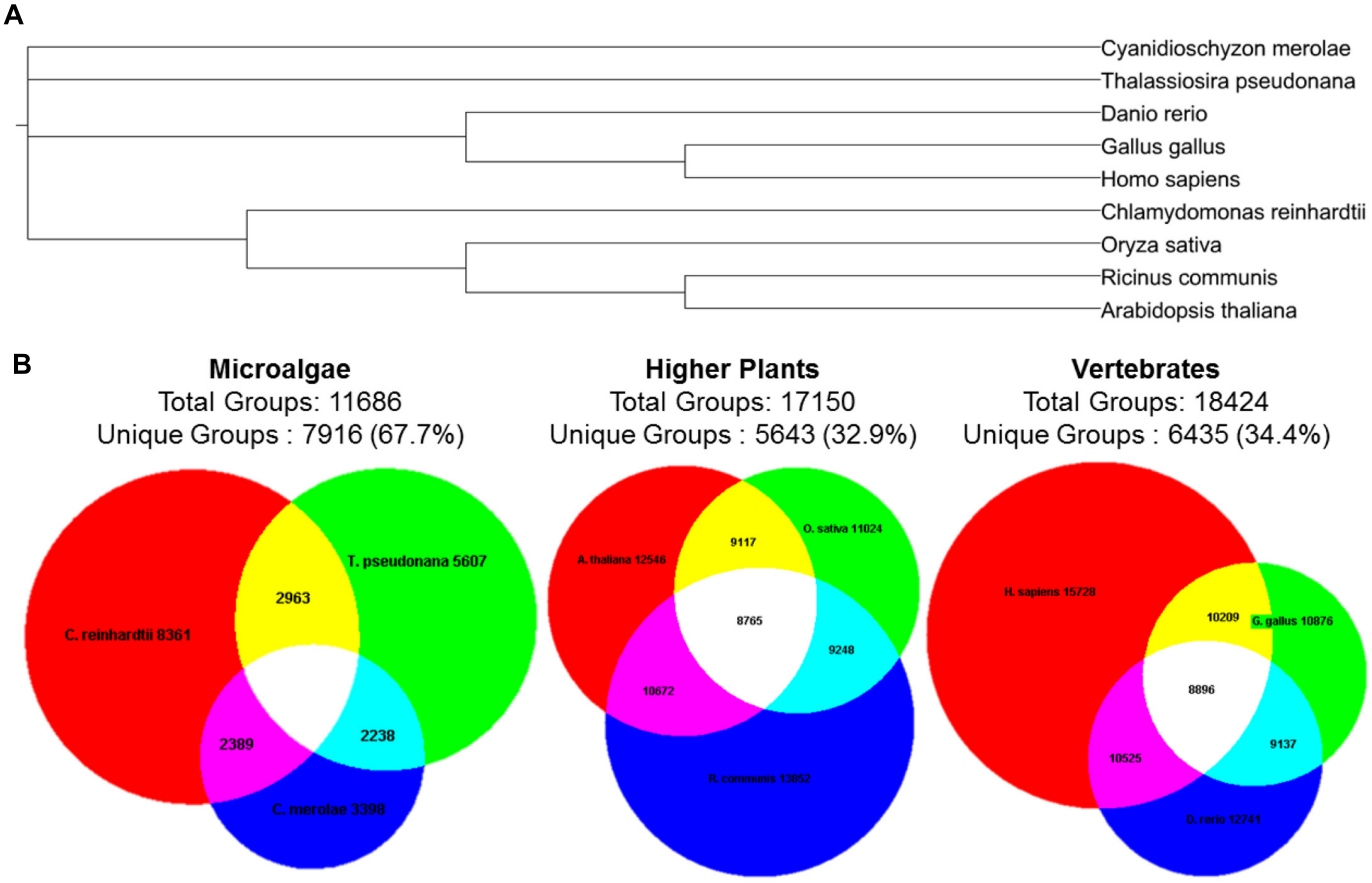
**Diversity of protein ortholog groups in microalgae, higher plants, and vertebrates. (A)** Phylogenetic tree of the selected species according to NCBI taxonomy. The tree was constructed using PhyloT and iTOL (Letunic and Bork, 2011). **(B)** Unique and shared groups of protein orthologs according to OrthoMCL database (Chen et al., 2006). The Venn diagrams were constructed using Euler3Applet (Chow and Rodgers, 2005). Common names for the species: *Chlamydomonas reinhardtii* (green alga, red circle); *Thalassiosira pseudonana* (diatom, green circle) *Cyanidioschyzon merolae* (red alga, blue circle); *Arabidopsis thaliana* (thale cress, red circle); *Oryza sativa* (rice, green circle); *Ricinus communis* (castor oil plant, blue circle); *Homo sapiens* (human, red circle); *Gallus gallus* (chicken, green circle); *Danio rerio* (zebrafish, blue circle).

Microalgae have evolved to adapt to a wide range of environments and consequently have proven to be a rich source of genetic and chemical diversity (Dufresne et al., 2008; Parker et al., 2008; Tirichine and Bowler, 2011; Blunt et al., 2012; Hildebrand et al., 2013). **Figure 1** shows that the polyphyletic nature of microalgae constitutes them as an excellent target for discovering unique groups of protein orthologs (Chen et al., 2006).

Furthermore, algae diversity has been exploited as a unique source of bioactive compounds like carotenoids, fatty acids (FAs), sterols, mycosporine-like amino acids, phycobilins, polyketides, pectins, halogenated compounds, toxins, etc., which have been extensively reviewed previously (Pulz and Gross, 2004; Spolaore et al., 2006; Cardozo et al., 2007; Hallmann, 2007; Milledge, 2011; Borowitzka, 2013; Leu and Boussiba, 2014). Currently, algae are the main sustainable source of commercial carotenoids and omega-3 FAs (Borowitzka, 2013; Leu and Boussiba, 2014). In addition, microalgae have also proven to be cost-effective and safe hosts for expressing a wide array of recombinant proteins, including human and animal therapeutics, and industrial enzymes (Specht et al., 2010; Gong et al., 2011b; Maliga and Bock, 2011; Rosales-Mendoza et al., 2012; Scranton et al., 2015). Furthermore, studies have shown that microalgae have also the potential to be an economically viable source of renewable biofuels (Stephens et al., 2010; Davis et al., 2011; Jones and Mayfield, 2011; Larkum et al., 2011).

While the potential of microalgae as a source of a wide range of products is high, optimization of cultivation and processing technologies will be required before algal-derived biofuels and some of the strain-specific biomolecules can be profitable on a large scale. Techno-economic analysis has identified that three main factors that significantly contribute to the overall cost of production for a microalgal metabolite are product content, growth rate, and cultivation cell density (Davis et al., 2011). These biological outputs are determined by a number of constraints including light intensity, nutrient supply, and the unique metabolism of individual species. Improvements in all these areas will be major drivers in creating the most efficient and economically viable strains of microalgae available for a diverse arrange of biotechnological applications. In this review we will mainly discuss strategies for increasing metabolite product content through genetic engineering. Nonetheless, metabolic engineering of photosynthesis and nutrient assimilation will also be discussed since they directly affect growth rate and cell culture density, thus overall metabolite productivity. Algae biomass can also be converted into biocrude and biogas, through thermochemical conversion and anaerobic digestion, respectively. Since these products do not depend on the accumulation of specific metabolites, engineering of photosynthesis and nutrient assimilation would also be a suitable approach for enhancing productivity of these types of fuels.

## Genetic Engineering of Microalgae

Microalgae can be transformed either in the nuclear, chloroplast or mitocondrial genomes (Specht et al., 2010). Most enzymes associated with secondary metabolism are coded in the nuclear genome, but some of them are targeted to the chloroplast for performing their function (Martin, 2010; Terashima et al., 2011; Heydarizadeh et al., 2013). In these cases either the nuclear or plastid genome could be engineered for a desired metabolic trait (Johanningmeier and Fischer, 2010). Electroporation, shaking with glass beads, and particle gun bombardment (biolistic) are the main methods for delivering DNA to microalgae (Coll, 2006), the latter has proven so far the only successful mean for chloroplast transformation (Purton et al., 2013). Nuclear transformation offers the advantages of post-translational modifications of the protein of interest, the possibility of protein targeting to any organelle, simpler transformation protocols and more flexible regulatory sequence recognition, allowing for the use of heterologous promoters and untranslated regions (Leon and Fernandez, 2007; Díaz-Santos et al., 2013; Scaife et al., 2015). It is worth mentioning that the nuclear genomes of some algae also contain efficient microRNA systems for gene silencing, which can also be harnessed for metabolic engineering (Molnar et al., 2009; Cerutti et al., 2011). On the other hand, the main disadvantage of nuclear transformation is the low expression levels of the genes of interest due to silencing and position effects (random integration; Leon and Fernandez, 2007; Specht et al., 2010; Scranton et al., 2015). Recently a system based on the co-translational fusion of an antibiotic-resistance gene with the gene of interest has proven to be effective for alleviating some of these drawbacks for nuclear expression in the model green alga, *Chlamydomonas reinhardtii* (Rasala et al., 2012). On the other hand, chloroplast gene expression offers higher levels of transgenic protein accumulation (usually ≥1% of total protein) and precise gene targeting given the effective homologous recombination machinery (Johanningmeier and Fischer, 2010; Purton et al., 2013). The latter also enables for efficient gene disruption for metabolic engineering purposes (Fischer et al., 1996). Then again, the disadvantages of plastid-based expression are the lack of post-translational glycosylation, lack of eukaryotic folding machinery, strict codon bias, and the requirement of the sequences for endogenous untranslated regulatory regions and homologous recombination flanking regions (Specht et al., 2010; Gimpel and Mayfield, 2013; Scaife et al., 2015; Scranton et al., 2015).

The great physiologic and genetic diversity of microalgae poses a great challenge for transforming new species given all the variables aforementioned. A proper DNA delivery system and optimized transformation conditions are recognized to be specific for each microalgal specie. Furthermore, the use of endogenous regulatory sequences has always proven to achieve the highest amount of transgenic protein accumulation, highlighting the need for endogenous genomic data (Díaz-Santos et al., 2013; Gimpel and Mayfield, 2013). Additionally, specie-specific codon optimization of the transgene is a critical condition for achieving detectable levels of transgenic protein accumulation (Specht et al., 2010; Gong et al., 2011b). Attempts of circumventing these requirements have resulted in non-reproducible transformation protocols. It is not rare to find past published data of algae transformation that has only been reported once, or by a single laboratory, even though the specie is a highly attractive transformation target for the scientific community (Chow and Tung, 1999; Teng et al., 2002; Coll, 2006; Hallmann, 2007; Chen et al., 2008).

## Engineering of the Lipid Synthesis Pathways

**FIGURE 2:**
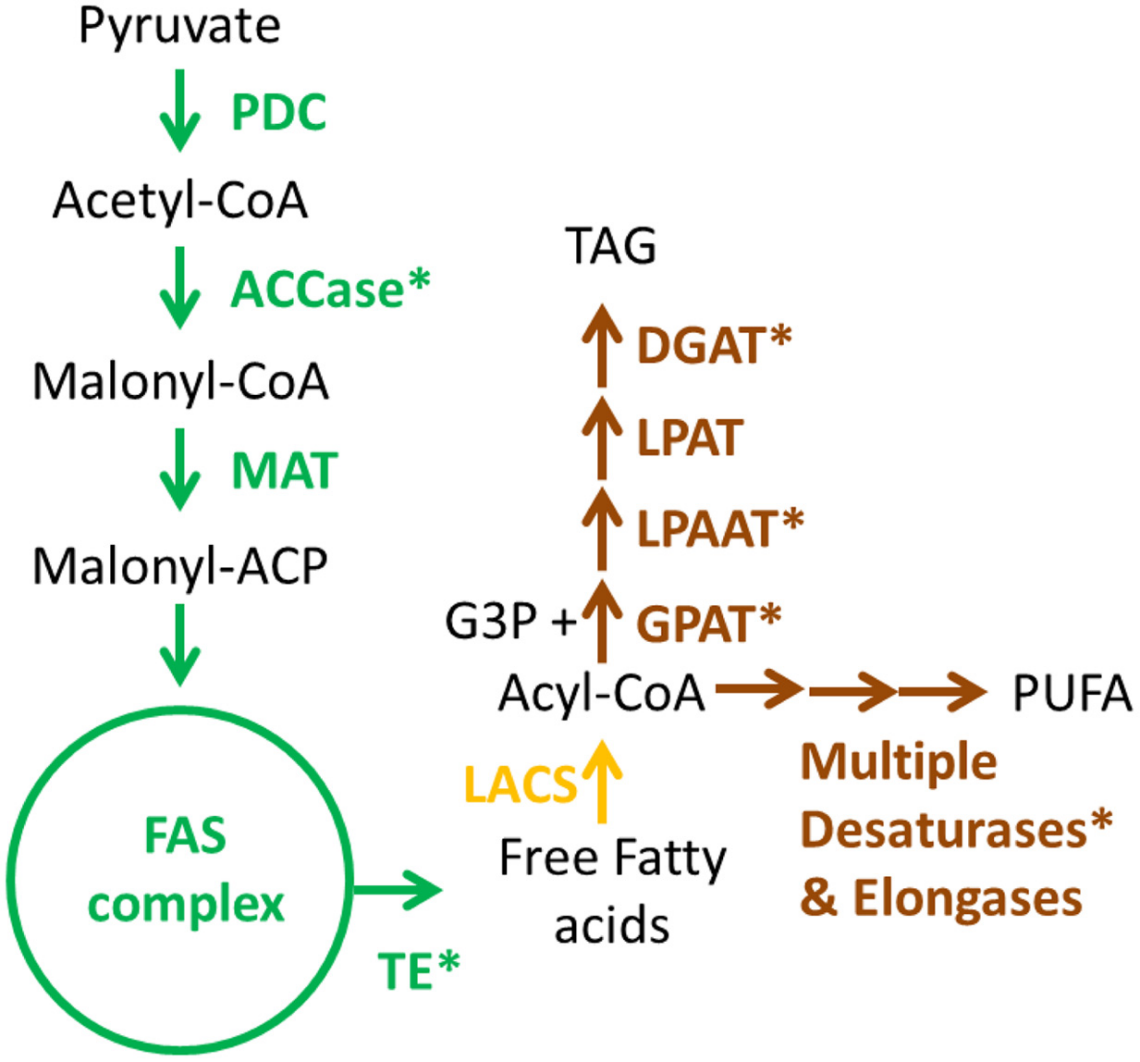
**Simplified biosynthetic pathways for fatty acids (FAs), triacylglycerols (TAGs) and polyunsaturated FAs in green microalgae.** Enzyme names are colored according to their localization. Green: plastid; Yellow: cytoplasm; Brown: endoplasmic reticulum. Asterisks denote enzymes which have been targeted for metabolic engineering, as shown in **Table 1**. PDC, pyruvate dehydrogenase complex; ACCase, acetyl-CoA carboxylase; MAT, malonyl-CoA/ACP transacylase; ACP, acyl-carrier protein; LACS, long-chain acyl-CoA synthetase; FAS, FA synthase; TE, fatty acyl-ACP thioesterase; GPAT, glycerol-3-phosphate acyltransferase; LPAAT, lyso-phosphatidic acid acyltransferase; LPAT, lyso-phosphatidylcholine acyltransferase; DGAT, diacylglycerol acyltransferase; CoA, coenzyme A; G3P, glycerate-3-phosphate; TAG, triacylglycerol; PUFA, polyunsaturated FA.

Lipid metabolism in microalgae can be regarded as complex and diverse given the metabolic divergence between the phylogenetic groups. It is clear that for engineering high-yield lipid producing strains, characterization of the specific pathways for any given phylogenetic group of algae will be required (Yu et al., 2011; Hildebrand et al., 2013; Bellou et al., 2014). A simplified scheme for FA, triacylglycerol (TAG), and polyunsaturated fatty acid (PUFA) biosynthesis in green algae is shown in **Figure 2**. Metabolic engineering strategies for these pathways are summarized in **Table 1**.

**Table 1:** Metabolic engineering reports for lipid synthesis in microalgae.

Target protein	Host	Type of modification	Gene source	Primary phenotype change	Reference
ACCase^∗^	*Cyclotella cryptica*	Nuclear oversexpression	Endogenous	No increase in total lipid accumulation	Dunahay et al., 1996
Acetyl-CoA carboxylase			*Navicula saprophila*		
PDK	*Phaeodactyllum tricornutum*	Antisense cDNA		82% increase in neutral lipids	Ma et al., 2014
Pyruvate dehydroganase kinase					
Malic enzyme	*Phaeodactyllum tricornutum*	Nuclear oversexpression	Endogenous	2.5-fold increase in total lipids	Xue et al., 2014
Multifunctional lipase	*Thalassiosira pseudonana*	RNAi		3.3-fold increase in total lipids	Trentacoste et al., 2013
TE^∗^ Fatty acid (FA)-ACP thioesterase	*Phaeodactyllum tricornutum*	Nuclear overexpression	Two higher plants	No increase in total lipid accumulation	Radakovits et al., 2011
				Increased production of C12 and C14 FA	
TE^∗^ Fatty acid-ACP thioesterase	*Phaeodactyllum tricornutum*	Nuclear overexpression	Endogenous	82% increase in total FAs	Gong et al., 2011a
				No change in chain-lengths profile	
TE^∗^ Fatty acid-ACP thioesterase	*Chlamydomonas reinhardtii*	Nuclear overexpression	Two higher plants	No increase in lipids for all genes	Blatti et al., 2012
			Endogenous	Shorter chain FA with the endogenous gene	
DGAT^∗^ Acyl-Coa:diacylglycerol acyltransferase	*Chlamydomonas reinhardtii*	Nuclear overexpression	Three endogenous	No increase in TAG accumulation	La Russa et al., 2012
				No changes in TAG profiles	
DGAT^∗^ Acyl-Coa:diacylglycerol acyltransferase		RNAi; three target genes		24 and 37% decrease in TAGs with two genes	Deng et al., 2012
				34% increase in TAGs with one gene	
Five TAG biosynthetic enzymes^∗^	*Chlorella minutissima*	Nuclear overexpression	2 yeasts	Twofold increase in TAGS with five genes	Hsieh et al., 2012
				No change with individual genes	
Lipid trigger transcription factor	*Chlamydomonas reinhardtii*	Nuclear overexpression	Endogenous	11% increased total extractable lipids	Yohn et al., 2012
Lipogenesis transcription factor	*Chlorella ellipsoidea*	Nuclear overexpression	Soybean	52% increase in total lipids	Zhang et al., 2014
Δ4 desaturase^∗^	*Chlamydomonas reinhardtii*	Nuclear overexpression microRNA	Endogenous	Increased accumulation of FA 16:4	Zäuner et al., 2012
				Decreased accumulation of FA 16:4	
Δ5 desaturase^∗^	*Phaeodactyllum tricornutum*	Nuclear overexpression	Endogenous	58% increased accumulation of EPA	Peng et al., 2014
				65% more neutral FA	

*Enzymes with asterisks are shown in **Figure 2**.*

The first metabolic engineering report for increasing FA production in algae consisted in the overexpression of the acetyl-CoA carboxylase gene (*ACCase*) from the diatom *Cyclotella cryptica*. *ACCase* codes for the enzyme that carboxylates acetyl-CoA to malonyl-CoA, the first committed step for FA synthesis. Expression vectors and transformation protocols were developed for *C. cryptica* and the diatom *Navicula saprophila*. A two–threefold increase in the level of ACCase activity was reported for the transformed diatoms, but no increase in FA accumulation was detected. However, no experimental data was presented for the increase of ACCase activity (Dunahay et al., 1996). Eighteen years later research efforts have been undertaken once again for redirecting carbon and reducing potential toward lipid biosynthesis. Pyruvate can be transformed to acetyl-CoA (substrate for ACCase) through an oxidative decarboxylation reaction catalyzed by the mitochondrial pyruvate dehydrogenase complex (PDC), which is deactivated through phosphorylation by pyruvate carboxylase kinase (PDK). In contrast, plastid PDC is not regulated by a PDK homolog. Using an antisense cDNA construct, PDK expression was knocked down in the diatom *Phaeodactylum tricornutum*, resulting in up to 82% total neutral lipid increase without changes in the lipid profile (Ma et al., 2014). Malic enzyme (ME) catalyzes the decarboxylation of malate into pyruvate, producing at the same time NADH and carbon dioxide. In addition to pyruvate, NADH is an essential source of reducing power for lipogenesis. Overexpression of the endogenous ME in *P. tricornutum* resulted in a 2.5-fold increase of total lipid accumulation under nutrient-replete conditions when compared to the control. This great increase didn’t affect growth rate significantly, although cell morphology changes were observed (Xue et al., 2014). Another way of enhancing lipid accumulation is preventing lipid catabolism. After analyzing transcriptomic data from the diatom *Thalassiosira pseudonana* under silicon-deplete conditions a multifunctional lipase gene was selected as a target for knockdown experiments. RNAi and antisense constructs were transformed; the latter resulted in up to 3.3-fold higher total lipid content than wild-type during the exponential growth phase. Interestingly, the knockdown strains didn’t have a slower growth rate than WT (Trentacoste et al., 2013).

In order to enhance biodiesel cold flow properties, the total amount of short-chain FA (12-14 carbons) has been increased by overexpressing two higher plant FA acyl-carrier protein thioesterases (TE) in *P. tricornutum*. 75–90% of the short-chain FA were incorporated into TAGs, but there was no significant increase of total lipid accumulation (Radakovits et al., 2011). On the other hand, an endogenous TE was expressed in *P. tricornutum* (*PtTE*), resulting in 72% increase of total FA without modification of the FA chain-lengths profile (Gong et al., 2011a). This achievement in total FA increase would have a high impact if it can be reproduced in other species. Furthermore, two TE from higher plants and one endogenous TE gene from *C. reinhardtii* were overexpressed in this green alga. Only the overexpression of the endogenous gene (*CrTE*) resulted in higher levels of short FA, while no increase of lipid accumulation was achieved. The latter study also focused in the importance of achieving appropriate protein–protein interactions between the components of the FA synthesis machinery in order to obtain favorable metabolic engineering results (Blatti et al., 2012).

In addition to the modification of FA synthesis, the glycerol acylation steps have also been engineered for improving TAG accumulation. Overexpression of three endogenous Acyl-CoA:diacylglycerol acyltransferases (DGAT) in *C. reinhardtii* (*CrDGAT2a*, *b*, and *c*) didn’t result in increased TAG accumulation or changes in TAG profiles (La Russa et al., 2012). RNAi silencing of *C. reinhardtii CrDGAT2-1 or CrDGAT2-5* resulted in 24 and 37% decrease in lipid accumulation, respectively. Surprisingly, silencing of *CrDGAT2-4* caused an increase of up to 34% total lipids. *CrDGAT2-2 or CrDGAT2-3* silencing didn’t cause significant changes in lipid accumulation (Deng et al., 2012). In contrast with the results of (La Russa et al., 2012), overexpression of the endogenous *DGAT2* in the diatom *P. tricornutum* resulted in 35% increase of neutral lipid accumulation without significantly affecting growth rate. Furthermore, the valuable omega-3 eicosapentaenoic acid (EPA) accumulated 76% more than in the control (Niu et al., 2013). The simultaneous expression of five TAG biosynthesis-related enzymes derived from the yeasts *Saccharomyces cerevisiae* and *Yarrowia lipolytica* (phosphatidic acid phosphatase, lysophosphatidic acid acyltransferase, glycerol-3-phosphate dehydrogenase, glycerol-3-phosphate acyltransferase, and DGAT) in the green microalga *Chlorella minutissima* resulted in a twofold increase in TAG accumulation. The expression of each enzyme by itself didn’t result in significant changes in lipid accumulation, thus pointing toward the effectiveness of system level approaches (Hsieh et al., 2012).

Transcription factors (TF) can also be manipulated as an alternative to “classic” genetic engineering, which could prove to be more effective for generating global metabolic changes. A patent from Sapphire Energy, Inc. describes that the overexpression of the SN03 TF produces the effect of a “lipid-trigger” in *C. reinhardtii*, thus resulting in 11% more accumulation of total extractable lipids in nutrient-replete medium. This TF was chosen based on comparative transcriptomic data between nutrient-replete and nitrogen-deplete cells (Yohn et al., 2012). Likewise, a lipogenesis promoting TF from soybean was overexpressed in the green alga *C. ellipsoidea*, which accumulated up to 52% more total lipids. This TF resulted in the differential expression of 1046 transcripts, including the up-regulation of ACCase (top six up-regulated transcripts were annotated as ACCase; Zhang et al., 2014).

Omega-3 polyunsaturated FAs from microalgae can reach much higher prices than biodiesel. Therefore commercial production of PUFA from genetically engineered algae could be a viable option in a much shorter term, but for a much smaller and more regulated market. Metabolic engineering of PUFA deals mostly with the overexpression of specific FA desaturases and elongases, although TE have also proven useful for this purpose (Niu et al., 2013). In *C. reinhardtii*, overexpression of the endogenous Δ4 desaturase (CrΔ4FAD) resulted in increased accumulation of its specific product hexadeca-4,7,10,13-tetraenoic acid (16:4). Alternatively, silencing through microRNA resulted in decreased 16:4 accumulation. This shows that classical genetic engineering approaches can result in rational results when modifying the expression of FA desaturases (Zäuner et al., 2012). An endogenous Δ5 desaturase (PtD5b) was overexpressed in *P. tricornutum* resulting in 58% increased accumulation of EPA. Additional poly and mono unsaturated FA were also significantly augmented. Moreover, this strain accumulated 65% more neutral FA, showing that this strategy could also serve for improving biodiesel yields (Peng et al., 2014).

The complexity of lipid biosynthesis poses a great challenge if the final goal is to enhance accumulation of not only a single type of lipid, but overall lipid accumulation for biofuels. It is then necessary to investigate in further details the regulatory mechanisms in order to deploy system level metabolic engineering of lipid biosynthesis. The studies from (Yohn et al., 2012), and (Zhang et al., 2014) involving TF engineering are great examples in this direction. Additionally there is a lack of efforts toward transforming lipogenic enzymes into the chloroplast genome, given that most of the initial reactions for FA synthesis take place in this organelle (**Figure 2**).

## Enhancement of Biohydrogen Evolution

Green microalgae from the genera *Chlamydomona*, *Scenedesmus*, *Lobochlamys*, and *Chlorella* can reduce protons to produce hydrogen gas due to their hydrogenase activity, determined to derive predominantly from the [FeFe]-hydrogenase (HYDA1 in *C. reinhardtii*; Meuser et al., 2009, 2012). Biohydrogen is an attractive fuel alternative because its combustion produces no carbon byproducts and it is a superior fuel for electricity production by fuel cells. Hydrogen production cannot be sustained while photosynthesis is actively occurring because oxygen inactivates hydrogenase. Therefore, a bi-phasic production strategy is necessary, in which algae grow photosynthetically to accumulate biomass, then the cells are exploited for H_2_ production under anoxic conditions, which can be achieved by inhibition of Photosystem II (PSII) by sulfur deprivation or by using herbicides (oxidation of water by PSII generates oxygen; Melis et al., 2007; Beer et al., 2009). Nonetheless, two novel strains of *C. vulgaris* have been shown to be able to produce hydrogen under atmospheric oxygen concentrations (Hwang et al., 2014). Increasing hydrogenase activity (or decreasing its oxygen sensitivity), anaerobiosis induction, avoiding competition for electrons from other pathways and increasing the sources of electrons are all steps that could be improved through genetic engineering (Melis et al., 2007; Beer et al., 2009; Esquível et al., 2011; Dubini and Ghirardi, 2014).

Engineering hydrogenase in order to reduce its oxygen sensitivity has yielded only modest improvements, primarily due to the fact that gas channels are formed when the catalytic site is properly folded (Dubini and Ghirardi, 2014). Alternatively, the endogenous hydrogenase has been overexpressed in *Chlorella* sp. strain DT, resulting in 7–10-fold hydrogen production under semi-aerobic conditions (Chien et al., 2012).

Inactivation of PSII in *C. reinhardtii* has been achieved by transforming an RNA antisense construct against a sulfate transporter gene (SulP), leading to the accumulation of hydrogen even in the presence of 100 μM sulfate (Chen et al., 2005). Another system for achieving anaerobiosis is based on a copper responsive nuclear transgene that is necessary for the expression of the essential protein D2 of PSII. Upon the addition of copper, the transgene is repressed, along with D2, thus stopping evolution of oxygen through PSII. A brief period of anaerobiosis and hydrogen production is achieved, but it is much shorter than the standard method through sulfur deprivation (Surzycki et al., 2007). In order to reduce PSII efficiency a double mutant of the reaction center protein D1 was generated. The resulting strain could evolve ten times more hydrogen than the control after sulfur deprivation, mainly due to a longer productive period (Torzillo et al., 2009). In *Chlorella* sp. strain DT expression knock-down of PsbO from PSII (part of the oxygen evolution center) through short interference antisense RNA resulted in up to 10-fold higher hydrogen evolution under semi-aerobic conditions (Lin et al., 2013). The Ligh-Harvesting Complex II (LHCII) captures and channels excitons toward PSII for photochemistry, and thus oxygen evolution. Knock-down of the three major proteins of *C. reinhardtii* LHCII (LHCMB1, 2, and 3) with three co-transformed RNAi constructs (each one specific for a single gene) resulted in a twofold increase in hydrogen production when compared to the parental strain (Oey et al., 2013).

Oxygen sequestration is another alternative for enhancing hydrogen biosynthesis. Chloroplast transformation of a codon-optimized leghemoglobin protein from soy, which sequesters oxygen in the nitrogen-fixing root nodules of this legume, along with a ferrochelatase from a nitrogen-fixing bacterium (for assembling the heme group) resulted in a fourfold increase of hydrogen production in *C. reinhardtii* (Wu et al., 2011). Alternatively, an *Escherichia coli* pyruvate oxidase has been expressed in the nucleus of *C. reinhardtii* in order to reduce intracellular oxygen concentration. This enzyme decarboxylates pyruvate into acetyl-phosphate while consuming one oxygen molecule. Transgenic algae strains produced up to 2.5-fold more hydrogen than the parental strain under very low light (30 μE m^-2^ s^-1^) and sulfur-replete conditions (Xu et al., 2011).

Securing additional reducing power can also result in increased hydrogen yields. The HUP1 hexose transporter from *C. kessleri* (green alga) was expressed in *C. reinhardtii*, resulting in a strain that could use glucose as a carbon and electron source (see Trophic Conversion below). This modification also translated into 1.5-fold higher hydrogen production rate (Doebbe et al., 2007). Carbon fixation by Rubisco competes for a significant amount of reducing power. Expression of a mutated small sub-unit of Rubisco (RBCS-Y67A) from the nucleus of an RBCS-deficient *C. reinhardtii* strain resulted in abolishment of PSII activity along with 10 to 15-fold increase in hydrogen yield in sulfur-deplete medium (Pinto et al., 2013). Ferredoxin-NADP^++^ reductase (FNR) is notoriously downregulated under sulfur deprivation in *C. reinhardtii*. RNAi knockdown of FNR resulted in decrease of Rubisco activity (60%) and oxygen evolution (44%), accompanied by increase in starch degradation (140%), under sulfur deprivation. These metabolic changes resulted in 2.5-fold increase in hydrogen production when compared to sulfur-starved WT (Sun et al., 2013).

Despite the limitations in short-term applicability of hydrogen based biofuels when compared to biodesel, algal hydrogen production has received a great amount of scientific attention. The research undergone so far constitutes *C. reinhardtii* as a model organism for hydrogen biogenesis. Advances in *C. reinhardtii* engineering could play a key role for developing more sustainable alternatives in the long-term, such as biohydrogen production from wastes degraded by either bacteria or other microalgae species.

## Metabolic Engineering of Carotenoids

**FIGURE 3:**
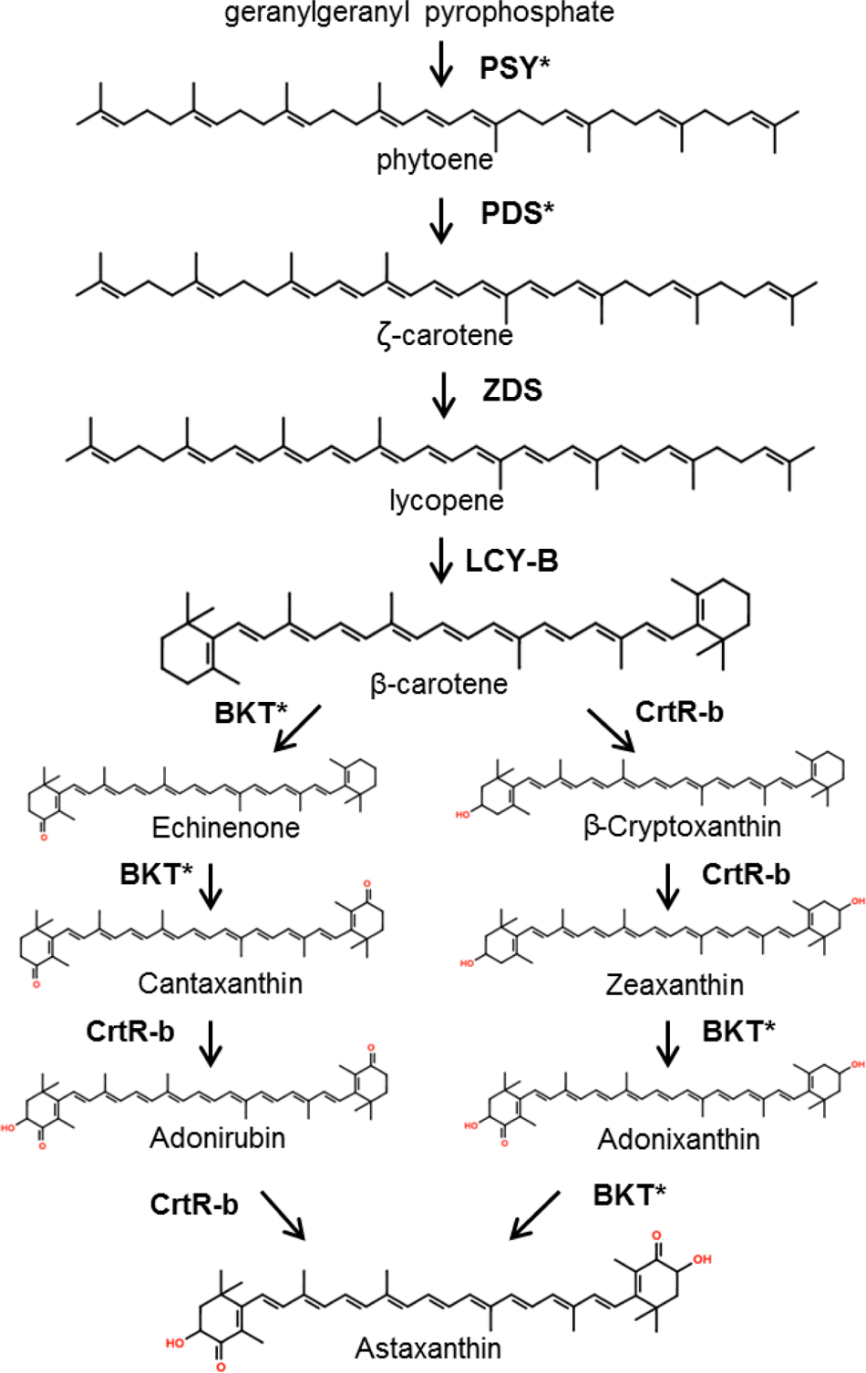
**Proposed pathway for astaxanthin synthesis in green microalgae.** Asterisks denote enzymes which have been targeted for metabolic engineering, as shown in **Table 2**. PSY, phytoene synthase; PDS, phytoene desaturase; ZDS, ζ-carotene desaturase; LYC-B, lycopene β-cyclase; BKT, β -carotene ketolase; CrtR-b, β-carotene hydroxylase. Modified from (Grünewald et al., 2001; Ye et al., 2008). Synthesis of β -carotene occurs in the chloroplast, while there is evidence that the subsequent steps could also take place in the cytoplasm (Grünewald et al., 2001; Guedes et al., 2011).

Despite the high value of carotenoids and the advantages of microalgal platforms, there have been few reported efforts toward carotenoid production optimization through metabolic engineering in these organisms. Since most of the carotenogenic pathway occurs in the chloroplast of algae, carotenoid metabolic engineering could be achieved by either nuclear or chloroplast transformation (or both; Kempinski et al., 2015). **Figure 3** shows the generally accepted pathway for β-carotene and astaxanthin biosynthesis in green microalgae. **Table 2** summarizes the metabolic engineering approaches for modifying terpenoid accumulation in these organisms. *C. reinhardtii* has served as the model organism for studying the effects of genetic engineering in carotenoid accumulation. In the first report of this type, an archeal heat-stable geranylgeranyl-pyrophosphate synthase (involved in the early steps of carotenoid biosynthesis) was expressed in the chloroplast of *C. reinhardtii*. Unfortunately, there were no measurable effects on the isoprenoid profile of the algae (Fukusaki et al., 2003). Three years later, another group attempted to produce keto-carotenoids (e.g., astaxanthin) in *C. reinhardtii* by nuclear overexpression of the beta-carotene ketolase genes from *H. pluvialis* (*bkt3*) and *C. reinhardtii* itself (*CRBKT*). Following several efforts using different expression vectors, no keto-carotenoids could be detected (Wong, 2006). In parallel, (León et al., 2007) followed an analogous approach, but using instead the *bkt1* gene from *H. pluvialis*. In this case a small peak of 4-keto-lutein could be detected, and it was not present in the parental strain. Unfortunately, no peak for astaxanthin was recorded. RNA interference technology has also been used for altering the carotenoid profile of *C. reinhardtii*. The phytoene desaturase gene (*pds*, coding for the second step of carotenoid biosynthesis) was targeted, resulting in a 93% reduction of its mRNA. Nonetheless, the carotenoid content didn’t change significantly, pointing toward the existence of additional rate-limiting processes (Vila et al., 2008). Additionally, the phytoene synthase gene (*psy*), which codes for the committing step enzyme for carotenoid synthesis, has been transformed in the *C. reinhardtii* nucleus causing an increase in carotenoid accumulation. Transformed strains overexpressing *psy* from *Dunaliella salina* and *C. zofingiensis* stored 2.6 and 2.2-fold more lutein than the wild-type, respectively (Cordero et al., 2011; Couso et al., 2011). Recently, the *C. reinhardtii* nucleus has been transformed with a point mutant version of its endogenous *pds* gene. The mutant enzyme had a 27% increase in its desaturase activity *in vitro*. The algae became resistant to the herbicide norflurazon and accumulated more lutein, beta-carotene, zeaxanthin, and violaxanthin *in vivo* (Liu et al., 2013).

**Table 2:** Metabolic engineering reports for terpenoid biosynthesis in microalgae.

Target protein	Host	Type of modification	Gene source	Primary phenotype change	Reference
Geranylgeranyl-pyrophosphate synthase	*Chlamydomonas reinhardtii*	Plastid overexpression	Archeabacterium	No changes in isoprenoid profile	Fukusaki et al., 2003
PSY^∗^ Phytoene synthase	*Chlamydomonas reinhardtii*	Nuclear overexpression	*Dunaliella salina*	2.6-fold increase in lutein	Couso et al., 2011
PSY^∗^ Phytoene synthase	*Chlamydomonas reinhardtii*	Nuclear overexpression	*Chlorella zofingiensis*	2.2-fold increase in lutein	Cordero et al., 2011
PDS^∗^ Phytoene desaturase	*Chlamydomonas reinhardtii*	RNAi		93% reduction of mRNA Insignificant changes in carotenoid profile	Vila et al., 2008
PDS^∗^ Phytoene desaturase	*Chlamydomonas reinhardtii*	Nuclear overexpression	Endogenous w/mutation	Increase of several carotenoids Norflurazon resistance	Liu et al., 2013
PDS^∗^ Phytoene desaturase	*Chlorella zofingiensis*	Nuclear overexpression	Endogenous w/mutation	32.1% increase in total carotenoids 54.1% increase in astaxanthin	Liu et al., 2014
PDS^∗^ Phytoene desaturase	*Haematococcus pluvialis*	Nuclear overexpression	Endogenous w/mutation	26% increase in astaxanthin Norflurazon resistance	Steinbrenner and Sandmann, 2006
PDS^∗^ Phytoene desaturase	*Dunaliella salina*	RNAi		72% reduction of mRNA Carotenoid profiles were not reported	Sun et al., 2008
BKT^∗^ β-carotene ketolase	*Chlamydomonas reinhardtii*	Nuclear overexpression	*Haematococcus pluvialis* Endogenous	No keto-carotenoids detected	Wong, 2006
BKT^∗^ β-carotene ketolase	*Chlamydomonas reinhardtii*	Nuclear overexpression	*Haematococcus pluvialis*	4-keto-lutein detected No astaxanthin detected	León et al., 2007
Squalene synthase	*Chlamydomonas reinhardtii*	Nuclear overexpression	Endogenous	Squalene was not detected	Kajikawa et al., 2015
Squalene epoxidase	*Chlamydomonas reinhardtii*	RNAi		56–76% knock-down of mRNA Up to 1.1 μg/mg DW of squalene	Kajikawa et al., 2015

*Enzymes with asterisks are shown in **Figure 3**.*

*Haematococcus pluvialis*, *D. salina*, and *Chlorella* sp. are highly relevant candidates for carotenoid metabolic engineering given their commercial relevance. In the first attempt of stable nuclear transformation, *H. pluvialis* was engineered with a mutant *pds* gene that conferred resistance to norflurazon. Transgenic strains accumulated up to 26% more astaxanthin than the wild-type control after 48 h of induction with high-light (Steinbrenner and Sandmann, 2006). RNAi constructs have been used for reducing the mRNA accumulation of the *D. salina pds* gene for up to 72%. Intriguingly, the carotenoid content of these strains was not reported (Sun et al., 2008). The most recent metabolic engineering effort involves the development of a nuclear transformation method for *C. zofingiensis*. A mutant version of the endogenous *pds* gene was transformed, conferring resistance to norflurazon. The mutant PDS enzyme had 33% higher desaturation activity *in vitro*. Transformed *C. zofingiensis* strains accumulated up to 32.1% more total carotenoids and 54.1% more astaxanthin *in vivo* (Liu et al., 2014).

It is worth mentioning that additional terpenoids from microalgae can also be regarded as highly desirable feedstocks for biofuels and specialty chemicals, which will further broaden the interest for research toward optimizing the production of these compounds (Davies et al., 2014; Heider et al., 2014). Recently, *C. reinhardtii* has been engineered for accumulating the highly valuable triterpenoid squalene. Wild-type *C. reinhardtii* contains the necessary genes for squalene synthesis, although it cannot be detected under standard growth conditions. Overexpression of the endogenous squalene synthase didn’t result in squalene accumulation. On the other hand, silencing of squalene epoxidase (56–76% mRNA reduction) resulted in accumulation of up to 1.1 μg/mg dry weight of squalene. Transformation of squalene synthase into these knockdown strains didn’t result in enhanced squalene accumulation (Kajikawa et al., 2015).

Overall, carotenoid metabolic engineering in microalgae has not yielded consistent results. Perhaps the targeted enzymes do not constitute the bottleneck steps, or there are several rate-limiting reactions in the pathway. The future trend in carotenoid engineering should be the simultaneous transformation of three or more enzymes to strengthen the desired metabolic flow. Discovery and engineering of microalgae TFs that regulate terpenoid synthesis should also be considered for this purpose. Use of herbicide resistant phytoene desaturase has proven useful for enhancing carotenoid accumulation (Liu et al., 2014). This system could also be expanded to additional terpenoid biosynthetic enzymes like deoxyxylulose 5-phosphate synthase, deoxyxylulose 5-phosphate reductoisomerase, phytoene synthase, ζ-carotene desaturase, and lycopene β-cyclase, which are sensitive to various bleaching herbicides (Sandmann, 2002; Ferhatoglu and Barrett, 2006).

## Robust Carbon Dioxide Fixation

When microalgae are grown under phototrophic conditions, all newly produced biomass, including lipids, derive from the fixation of CO_2_ into ribulose-1,5-biphosphate (RuBP) to form 3-phosphoglycerate, catalyzed by the famous enzyme RuBP carboxylase/oxygenase (Rubisco). Additional enzymes are also required to regenerate RuBP in a process named the Calvin–Benson–Bassham cycle (CBB). Significant amounts of ATP and NADPH are also required for this process, which are supplied by the light-driven activity of photosystems I and II (photochemistry; Raines, 2011). Several studies have shown that the activity of Rubisco is the major bottleneck for carbon flux through the CBB when CO_2_ is not supplied to the media, or under high-light (excess ATP and NADPH) or high-temperature conditions (prevalence of photorespiration, see below), which are all present in commercial scale ponds for algae biomass production in desert areas (Raines, 2011; Ducat and Silver, 2012).

Rubisco is regarded as a “slow and confused” enzyme. Large amounts of this enzyme are required for achieving a sustainable carboxylation rate and it has affinity for oxygen which is used in a futile reaction. The affinity toward oxygen increases at higher temperatures. The consequences of the wasteful oxygenation reaction are partially alleviated by a process named photorespiration (Whitney et al., 2011). *C. reinhardtii* seems to be a perfect host for engineering Rubisco, since there are Rubisco deficient strains that can complete their life cycle heterotrophically, unlike plants (Whitney et al., 2011). The small subunit of Rubisco genes (*rbcS*) of *Arabidopsis* and sunflower have been transformed independently into an *rbcS* deficient strain of *C. reinhardtii* (lacking the two nuclear *rbcS* alleles), while preserving the endogenous large subunit gene in the chloroplast (*rbcL*). The *in vitro* CO_2_/O_2_ specificity or discrimination factor (Ω) was improved by up to 11% while maintaining the *V*max of carboxylation catalysis (*V*c). Nonetheless the cells displayed slower autotrophic growth rates and lacked pyrenoids (carbon-concentration sub-compartments in the algal chloroplast), presumably due to mistargeting of Rubisco caused by the heterologous small subunits (Genkov et al., 2010). *Chlamydomonas rbcL* has also been subjected to PCR-based gene shuﬄing with oligonucleotides representing the natural diversity of this gene. Three rounds of gene shuﬄing and three rounds of strain selection resulted in a Rubisco with increments of 20% Ω and 56% *V*c. Some of the enriched mutations were then incorporated into the tobacco *rbcL* gene and resulted in 14% Ω and 15% *V*c increments (Zhu et al., 2010). Another interesting strategy could consist in tuning Rubisco accumulation according to the environmental culture conditions in order to optimize the utilization of energy, carbon and nitrogen. *Chlamydomonas* strains with different amounts of Rubisco have been engineered by expressing the *rbcL* mRNA maturation factor MRL1 at different levels in the nuclear genome of an MRL1 deficient strain. Rubisco could be lowered up to 15% that of wild-type while maintaining phototrophic growth. An inducible promoter for MRL1 could be then used to tune Rubisco accumulation according to culture conditions, such as light intensity or CO_2_ concentration (Johnson, 2011).

The CBB cycle has also been engineered for enhancing carbon fixation. The CBB enzyme sedoheptulose 1,7-bisphosphatase enzyme (part of CBB) from *C. reinhardtii* has been overexpressed in β–carotene-producing green algae *D. bardawil*, resulting in a clear increase of oxygen evolution efficiency O_2_/cell/(μmol photons/m), and in organic carbon content per cell (Fang et al., 2012).

The future trends in Rubisco engineering should consider integrating the advances achieved in both RbcS and RbcL in the same microalgae strain. Future mutational studies should be performed in both genes simultaneously in order to capture co-variations that further enhance functionality. Additionally, there are two wide fields that have not been exploited in eukaryotic microalgae: the engineering of Rubisco activase and the carbon concentrating mechanisms.

## Trophic Conversion

Growing microalgae under heterotrophic or photoheterotrophic (mixotrophic) conditions has several advantages over autotrophic growth. Fermentation systems have been widely studied and successfully applied in industry for several years. The culture conditions are highly controlled and reproducible. Moreover, heterotrophic cultures of microalgae achieve higher cell densities, thus resulting in lower harvesting costs. In waste water treatment applications, trophic conversion allows to diversify the nutrients that can be degraded by algae (Chen and Chen, 2006; Chen et al., 2011). In addition, the yield of some metabolites can sometimes increase depending on the rerouting of the metabolic networks involved, as it has been shown for lipid accumulation (Miao and Wu, 2006). However, many microalgae species are strict autotrophs or are highly selective for their organic carbon source (e.g., acetate for *C. reinhardtii*). Trophic conversion has been achieved, allowing heterotophy in previously obligate phototrophic species as a proof of concept for simple metabolic engineering. *V. carteri* has been transformed with a hexose transporter from *C. kessleri* (*HUP1*, monosaccharide-H+ symporter), resulting in a strain that can survive on glucose under dark conditions (Hallmann and Sumper, 1996). *P. tricornutum*, *Cylindrotheca fusiformis* (diatoms) and *C. reinhardtii* have also been successfully transformed with *HUP1*, resulting in glucose transport into the cells (Fischer et al., 1999; Zaslavskaia et al., 2001; Doebbe et al., 2007). The human *glut1* transporter gene (erythrocyte glucose transporter 1) has also been transformed into *P. tricornutum*, which could also perform glucose uptake into the cell (Apt et al., 2011). It is worth noting that despite glucose incorporation, the extent of conversion to full heterotrophy is variable between these four algae.

Trophic conversion is a good proof of concept for microalgae metabolic engineering, but despite the advantages of heterotrophic culture, it might not to be optimal for the production of low-value metabolites (e.g., biodiesel). Additional costs for adding a carbon source and the requirement for enclosed bioreactors (given the higher risk of contamination) are major drawbacks compared to phototrophic systems. Furthermore, some metabolites accumulate upon the presence of high-light (e.g., carotenoids), excluding the possibility of saving costs associated with illumination.

## Photochemistry Optimization

Microalgae have evolved large light-harvesting complexes (LHC) for maximizing light absorption in low-light environments, where they naturally occur. Under artificial culture conditions (saturating light) excess energy is dissipated through heat and fluorescence quenching in the LHC. Excess energy that cannot be dissipated usually results in direct photodamage and the production of reactive oxygen species (photoinhibition). The large size of the LHC also limits light penetration into the culture medium, therefore lowering the maximum cell density that can be achieved in large scale facilities (Ort et al., 2011; Wobbe and Remacle, 2015). In the first genetic engineering attempt to overcome this, a single RNAi construct was effectively used for silencing all twenty LHC protein isoforms of *C. reinhardtii*. These cells have lower mRNA (0.1–26% relative to the control) and protein accumulation for all LHC genes and 68% less chlorophyll than the parental strain, resulting in 290% higher light transmittance in the culture. Furthermore, they present less dissipation energy through fluorescence quenching, which leads to an increase in photosynthetic quantum yield. Under high-light conditions, transformed cells were less susceptible to photoinhibition and grew at a 65% faster rate; however, they did not reach a higher cell density (Mussgnug et al., 2007). Later, the same research group achieved similar results by downregulating LHC expression at the translational level. NAB1 is a translation repressor of the LHCBM family (LHCII; Mussgnug et al., 2005), and its activation is redox dependent (Wobbe et al., 2009). A constitutively activated version of NAB1 (two amino acid mutations) was overexpressed in *C. reinhardtii*, resulting in a similar phenotype to the one obtained through RNAi. However, the effects were less dramatic, having a chlorophyll/cell reduction of 20% compared to that of 68% using RNAi, and a growth rate increase of 53% compared to the previous 65% (Beckmann et al., 2009). Even so, overexpression of a single repressor would be easier to reproduce in the future than silencing twenty LHC isoforms at the same time. Another group has worked with the *TLA1* gene (*truncated light-harvesting antenna 1*) of *C. reinhardtii*. Overexpression and silencing (RNAi) of *tla1* resulted in 13% increase and 70% reduction of chlorophyll/cell, respectively. This confirms that *tla1* is an attractive target for modifying phototrophic growth performance of algae (Mitra et al., 2012). As previously mentioned in the biohydrogen section of this review, the three major proteins of *C. reinhardtii* LHCII (LHCMB1, 2, and 3) have been knocked-down with three co-transformed RNAi constructs (each one specific for a single gene). This resulted in 50% reduction of chlorophyll/cell, and four times more light penetration at equal cell density. Additionally, the transformed strain grew 85% faster at a 5 mm culture depth under 450 μE m^-2^ s^-1^ light intensity (Oey et al., 2013).

One of the main bottlenecks for photosynthetic growth under suboptimal conditions is PSII, the multi-protein complex that performs the light-driven oxidation of water. Degradation of the D1 sub-unit of PSII seems to be predominantly increased when light is in excess or under various abiotic stresses (photoinhibition; Kreslavski et al., 2007; Keren and Krieger-Liszkay, 2011). Rea et al. (2011) have mutated and selected versions of the *C. reinhardtii* D1 protein that can evolve up to ∼4.5-fold more oxygen *in vivo* under high-light conditions (50% midday sunlight) compared to the control. This has been achieved by transforming error-prone PCR-amplified D1 coding sequences followed by selection under ionizing radiation. Unfortunately the mutant strains perform slightly worse under laboratory light conditions (10% midday sunlight), suggesting that this strategy may not translate to increased biomass yield for commercial biofuel production. The cyanobacteria *Synechoccocus* sp. PCC 7942 contains two isoforms of the D1 protein which are differentially expressed under low-light and high-light conditions. These two proteins were expressed independently in *C. reinhardtii* resulting in reconstitution of the low-light and high-light phenotypes associated with each D1 isoform. Interestingly, *C. reinhardtii* expressing the cyanobacterial low-light isoform yielded 11% more dry weight biomass than the strains expressing the high-light isoform or the endogenous D1 protein, which is a highly desirable for reducing harvesting costs (Vinyard et al., 2013). Using *C. reinhardtii* as a transformation host, the same authors determined the precise amino acids that confer the characteristic phenotypes to these two D1 isoforms. The latter will prove very important for designing strategies to optimize D1 and PSII function under specific growth conditions (Vinyard et al., 2014).

In coming years, engineering of the photosynthetic machinery should take into account the adverse environmental conditions that can take place in outdoor ponds, such as high and low irradiance, and very high temperatures, which can all decrease photosynthetic rates (Kreslavski et al., 2007; Keren and Krieger-Liszkay, 2011). Tuning photosystems and light harvesting antennas to perform under specific environmental conditions, or even better, generating regulated systems that can adapt to environmental changes, would be major breakthroughs for large-scale algae cultivation. Furthermore, these advances could potentially be translated to commercial crops. In order to achieve this, researchers will have to consider that the photosynthetic machinery is composed of large complexes of highly interacting proteins. Any major advancement will likely require the engineering of several of these proteins simultaneously, in order to maintain the interactions that have been conserved through the extended evolutionary history of photosynthetic systems.

## Future Perspectives

Highly predictive metabolic models will be required in order to step-up metabolic engineering of microalgae (Veyel et al., 2014). At least eleven genome-wide metabolic network models are available for microalgae, but most of them correspond to *C. reinhardtii*, even though there are more than 30 sequenced species (Reijnders et al., 2014; Baroukh et al., 2015). Given the great metabolic diversity of microalgae, it is clear that models for at least each phylum will be required in order to give meaningful predictions for the corresponding species within that group (Hildebrand et al., 2013). It is also worth mentioning that metabolic engineering of algae doesn’t have to be circumscribed to metabolites that already exist in these organisms. For example *C. reinhardtii* has been engineered to produce the five-carbon sugar-alcohol xylitol, an artificial sweetener that doesn’t naturally occur in the alga. Xylose reductase (XR) from the filamentous fungus *Neurospora crassa* was codon-optimized and expressed in the plastid genome resulting in up to 0.38 g/L xylitol accumulation (Pourmir et al., 2013).

**FIGURE 4:**
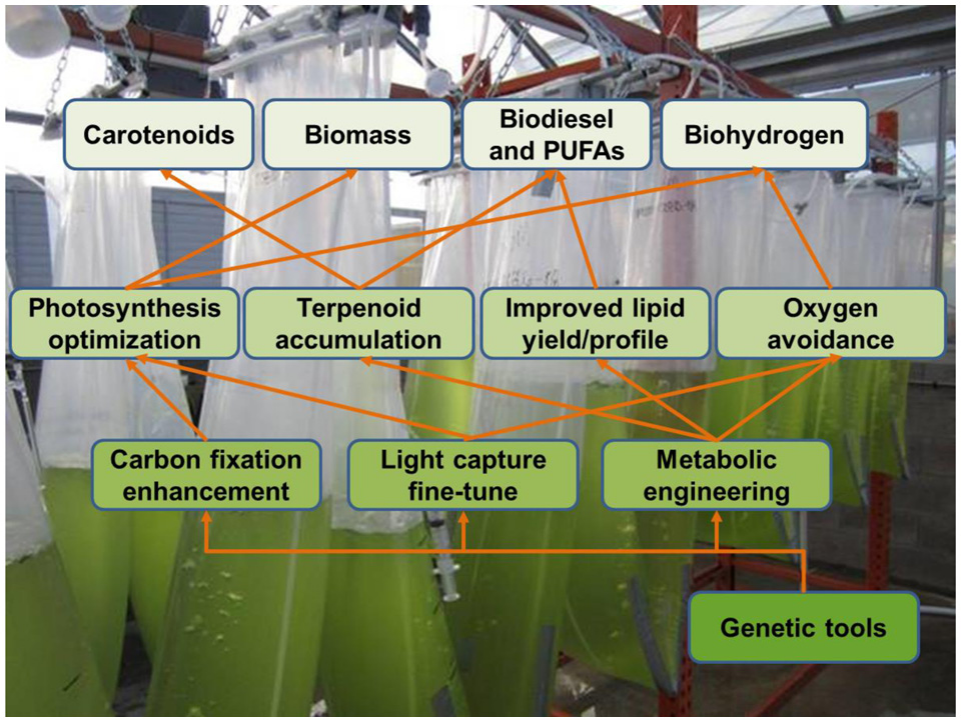
**Contribution of microalgal genetic tools to enhance the production of secondary metabolites and biomass**.

Novel genetic tools will also be a major driving force for metabolic engineering in eukaryotic microalgae (**Figure 4**). Nuclear genome editing would allow for precise gene deletion and gene integration, therefore enabling to reroute metabolic networks and to obtain predictable expression levels of transgenes (Jinkerson and Jonikas, 2015). Unfortunately, homologous recombination rates in *C. reinhardtii* are very low, but there are ongoing efforts to circumvent this through zinc-fingers technology (Sizova et al., 2013), and by using the CRISPR/Cas9 system (Jiang et al., 2014). On the other hand, some microalgae appear to have a highly efficient homologous recombination machinery in the nucleus, like for example *Nannochloropsis* sp. strain W2J3B (Kilian et al., 2011). Targeted induction or constitutive activation of endogenous genes is another valuable tool for modifying metabolic profiles. Activation of endogenous genes through transcription activator-like effectors (TALE) has been achieved in *C. reinhardtii* for the nuclear genes coding for arylsulfatase (ARS, endogenous colorimetric reporter), and the inorganic carbon membrane transporter HLA3 (Gao et al., 2014, 2015). Chloroplast metabolic engineering could take great advantage of a “shuttle” chloroplast genome that can replicate in *C. reinhardtii*, *S. cerevisiae*, and *E. coli* (O’Neill et al., 2012). Replication in *S. cerevisiae* allows for extensive DNA manipulations through gene replacement which would be required for simultaneous engineering of multiple enzymes in a metabolic pathway. *E. coli* replication serves for generating multiple copies of the genome for algae transformation (O’Neill et al., 2012). This “shuttle genome” strategy could also be complemented by a set of synthetic 5′ UTR for *C. reinhardtii* plastid, which could tune the expression of the multiple components of an engineered pathway (Specht and Mayfield, 2012). Recently, the diatoms *P. tricornutum* and *T. pseudonana* have been transformed with DNA episomes that replicate independently in the nucleus. These constructs carry a yeast-derived maintaining region that allows for stable replication even in the absence of antibiotic selection. Furthermore, these vectors can be transformed by trans-kingdom conjugation between *E. coli* and the diatoms (Karas et al., 2015). These results would allow for gene expression without position effects, and from multiple gene copies. If it is easily reproducible, conjugative transformation would eliminate the requirement for expensive biolistic or electroporation equipment for diatom transformation, thus enabling more laboratories to work in these organisms.

## Conflict of Interest Statement

The authors declare that the research was conducted in the absence of any commercial or financial relationships that could be construed as a potential conflict of interest.
